# Supplementary material for: Multi-Perspective Observation on the Prevalence of Food Allergy in the General Chinese Population: A Meta-Analysis
Source: Nutrients. 2022 Dec 6;14(23):5181. doi: 10.3390/nu14235181 (PMC9736235; doi:10.3390/nu14235181)
Supplement: Supplementary file 1 [file nutrients-14-05181-s001.zip › nutrients-1985773-supplementary.pdf]

Supplementary material

Table S1 JBI Checklist for Prevalence Studies

Study	Q1	Q2	Q3	Q4	Q5	Q6	Q7	Q8	Q9
Hu et al., 2021	Yes	Yes	Yes	Yes	Yes	No	Unclear	Yes	Yes
Tang et al., 2021	No	Unclear	Yes	Yes	No	No	Yes	Yes	Yes
Xu et al., 2020	Yes	Yes	Yes	Yes	Unclear	No	Yes	No	Yes
Sun et al., 2020	Yes	Yes	Yes	No	Unclear	No	Unclear	Yes	Yes
Zang et al., 2019	Yes	Yes	Yes	No	Yes	No	Unclear	Yes	Yes
Yu et al., 2019	Yes	Yes	Yes	Yes	Yes	No	No	Yes	Yes
Wang et al., 2017	Yes	Yes	No	Unclear	Yes	No	Yes	Yes	Yes
Zhang et al., 2016	No	Yes	Unclear	Yes	Yes	No	Yes	Yes	Yes
Wang et al., 2016	Yes	Yes	Yes	Yes	Yes	No	Yes	Yes	Yes
Chen et al., 2015	Yes	Yes	Yes	Yes	Yes	No	Yes	Yes	Yes
Ye et al., 2010	Yes	Unclear	Yes	No	Unclear	No	Unclear	Yes	Yes
Feng et al., 2021	Yes	Yes	Yes	No	Unclear	No	Yes	Yes	Yes
Li et al., 2019	Yes	Yes	Yes	Yes	Yes	No	Yes	Yes	Yes
Shao et al., 2017	Yes	Yes	Yes	Yes	Yes	No	Yes	Yes	Yes
Xie et al., 2017	Yes	Yes	Yes	Yes	Yes	No	Yes	Yes	Yes
Ma et al., 2009	No	Yes	Yes	Yes	Yes	No	Yes	Yes	Yes
Wang et al., 2019	Yes	Unclear	Yes	Yes	Yes	No	Yes	Yes	Yes
Zeng et al., 2015	Yes	Yes	Yes	Yes	Yes	No	Yes	Yes	Yes
Zhang et al., 2015	Yes	Yes	No	Yes	Unclear	No	Yes	Yes	Yes
Liu et al., 2013	Yes	Yes	No	Yes	Yes	No	Yes	Yes	Yes
Mo et al., 2013	Yes	Yes	Yes	No	No	No	Unclear	Yes	Yes
Wang et al., 2013	Yes	Yes	Yes	No	Unclear	No	Yes	Yes	Yes
Wu et al., 2012	Yes	Yes	Yes	Yes	Yes	No	Yes	Yes	Yes
Leung et al., 2009	Yes	Yes	Yes	Yes	Yes	No	Yes	Yes	Yes
Zhang et al., 2021	No	Unclear	Yes	Yes	Unclear	Yes	Yes	Yes	Yes
Pan et al., 2020	Yes	Yes	Yes	Yes	Unclear	Yes	Yes	Yes	Yes
Li J et al., 2019	Yes	Yes	Yes	Yes	Yes	Yes	Yes	Yes	Unclear
Yang et al., 2015	Yes	Yes	Yes	Yes	Yes	Yes	Yes	Yes	Unclear
Jin et al., 2012	No	Unclear	Yes	Yes	Yes	Yes	Yes	Yes	Yes
Lv et al., 2005	No	No	Yes	No	Yes	Yes	Yes	Yes	Yes
Huan et al., 2020	Yes	Yes	Yes	Yes	Yes	Yes	Yes	Yes	Yes
Peng et al., 2020	No	Yes	Unclear	No	Yes	Yes	Yes	Yes	Yes
Xiao et al., 2018	Yes	Yes	Yes	No	Unclear	Yes	Yes	Yes	Yes
Nie et al., 2017	No	No	Yes	Yes	Yes	Yes	Yes	Yes	Yes
Zou et al., 2013	Yes	Yes	Yes	Yes	Yes	Yes	Yes	Yes	Yes
Ma et al., 2020	No	No	No	Yes	Yes	Yes	Yes	Yes	Yes
Chen et al., 2012	No	No	Yes	Yes	Yes	Yes	Yes	Yes	Yes
Chen et al., 2011	No	No	Yes	Yes	Yes	Yes	Yes	Yes	Yes
Wei et al., 2022	Yes	Yes	Yes	Yes	Yes	No	Unclear	Unclear	Yes
Gou et al., 2022	Yes	No	Yes	Yes	Yes	Yes	Unclear	Unclear	Yes

Sun et al., 2022	No	Yes	Yes	Yes	Unclear	Unclear	No	Yes	Yes

The JBI Checklist provides quality criteria for prevalence studies in nine distinct items. The table indicates which items have been fulfilled for each included study, respectively:

Q1: Was the sample frame appropriate to address the target population?

Q2: Were study participants recruited in an appropriate way?

Q3: Was the sample size adequate?

Q4: Were the study subjects and setting described in detail?

Q5: Was data analysis conducted with sufficient coverage of the identified sample?

Q6: Were valid methods used for the identification of the condition?

Q7: Was the condition measured in a standard, reliable way for all participants?

Q8: Was there appropriate statistical analysis?

Q9: Was the response rate adequate, and if not, was the low response rate managed appropriately?

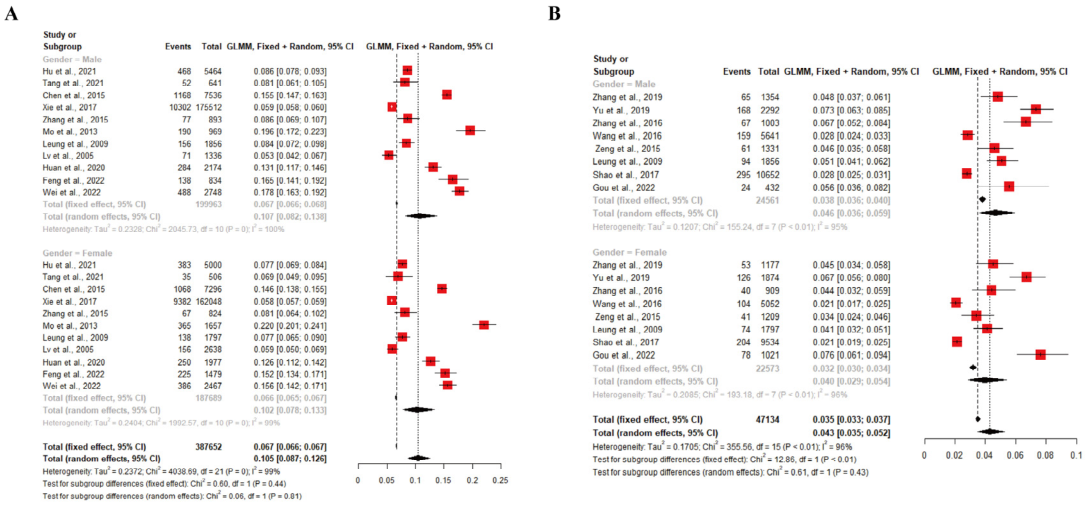

Figure S1 Forest plot for subgroup analyses of pooled prevalence of self-reported FA (A) and self-reported physician-diagnosed FA (B) based on gender.

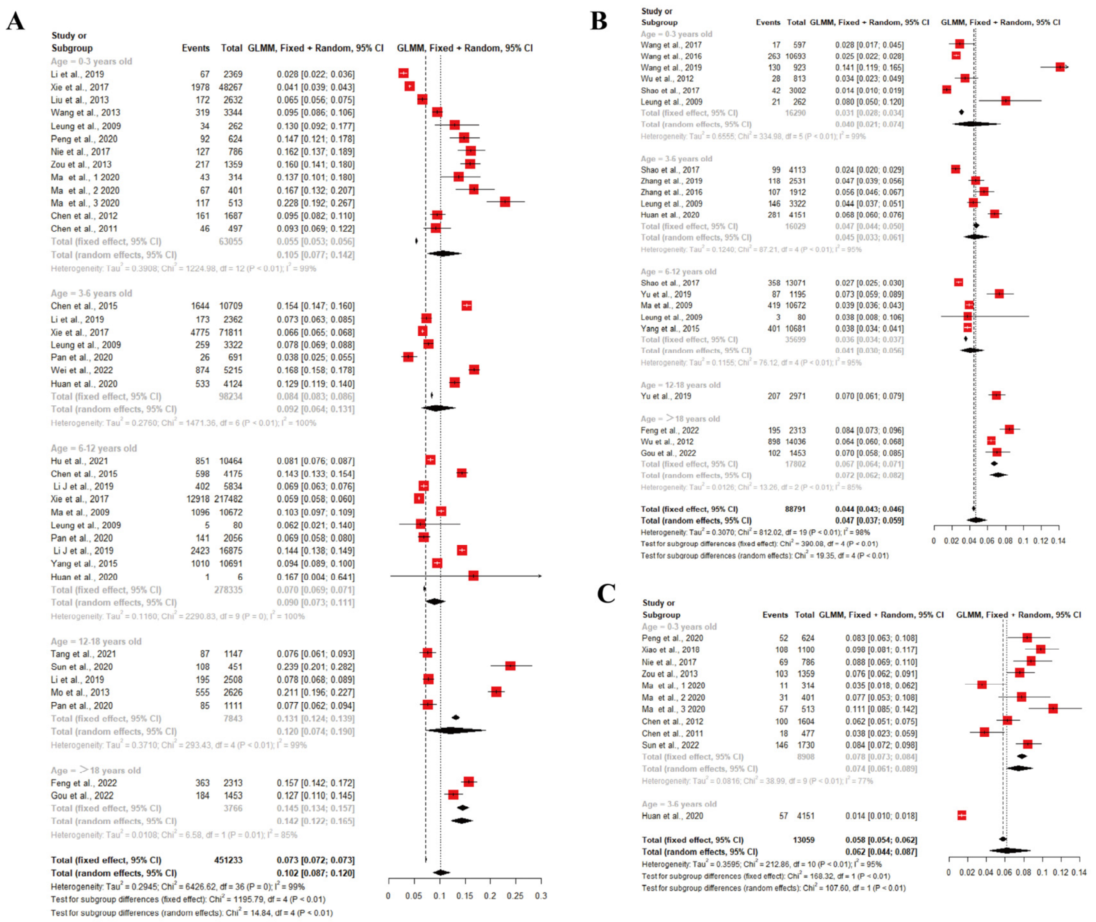

Figure S2 Forest plot for subgroup analyses of pooled prevalence of self-reported FA (A), self-reported physician-diagnosed FA (B), and OFC-positive FA (C) based on age.

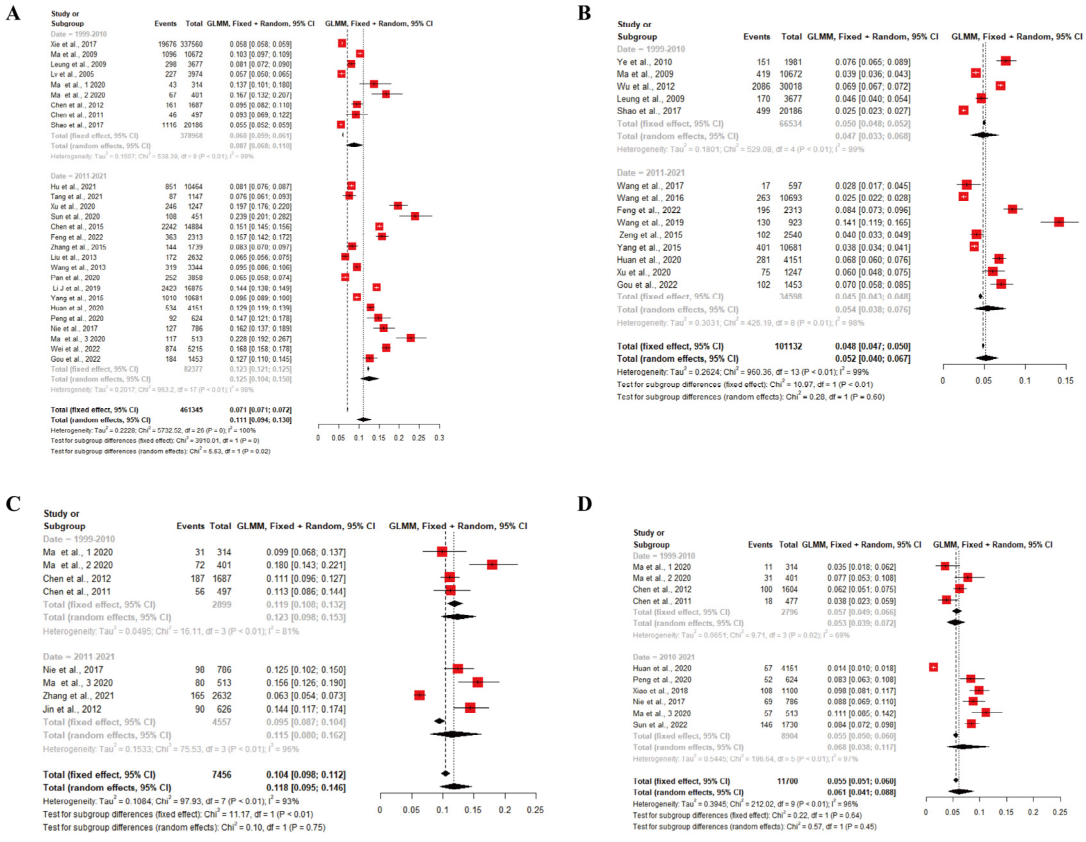

Figure S3 Forest plot for subgroup analyses of pooled prevalence of self-reported FA (A), self-reported physician-diagnosed FA (B), SPT-positive FA (C) and OFC-positive FA (D) based on year of data collection.

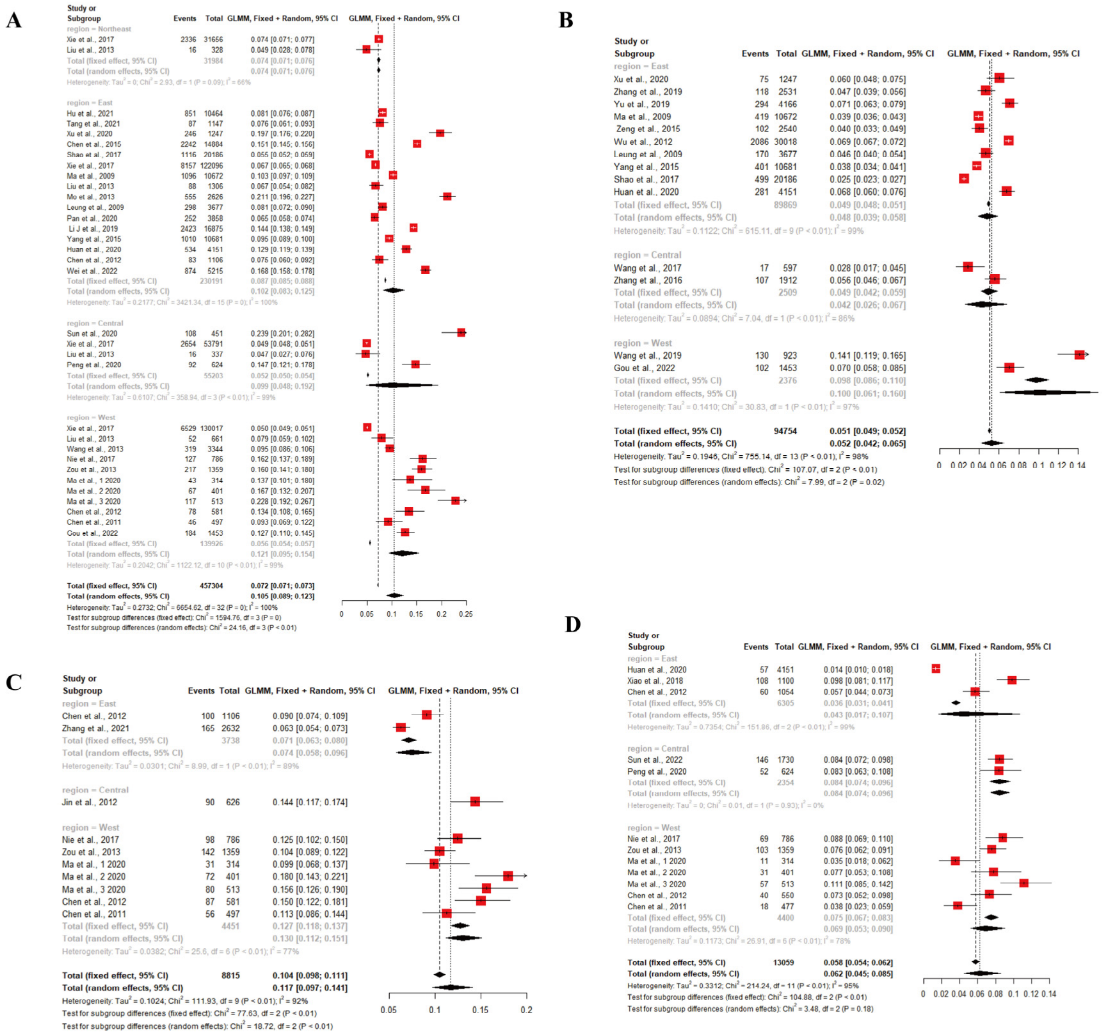

Figure S4 Forest plot for subgroup analyses of pooled prevalence of self-reported FA (A), self-reported physician-diagnosed FA (B), SPT-positive FA (C) and OFC-positive FA (D) based on region.

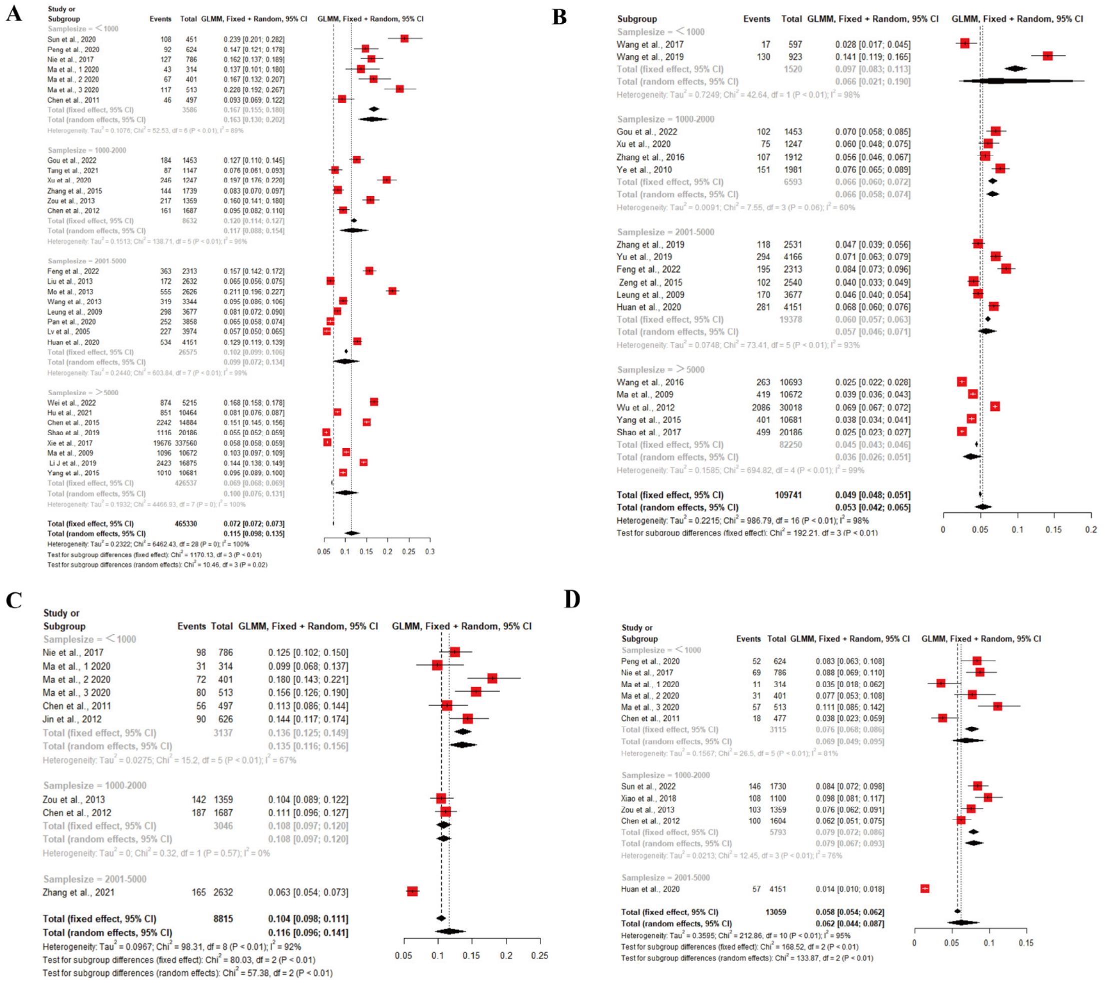

Figure S5 Forest plot for subgroup analyses of pooled prevalence of self-reported FA (A), self-reported physician-diagnosed FA (B), SPT-positive FA (C) and OFC-positive FA (D) based on sample size.

Table S2 meta-regression analyses for lifetime prevalence of self-reported FA in China

Covariate	Meta-regression estimate	95% CI	P value
Gender	-0.0501	-0.4627 to 0.3626	0.8119
Age	0.0436	-0.1001 to 0.1872	0.5523
Year of data collection	0.4033	0.0515 to 0.7551	0.0246
Regions	0.1397	-0.0384 to 0.3178	0.1242
Sample size	-0.1830	-0.3266 to -0.0394	0.0125

Table S3 meta-regression analyses for lifetime prevalence of self-reported physician-diagnosed FA in China

Covariate	Meta-regression estimate	95% CI	P value
Gender	-0.1688	-0.5834 to 0.2458	0.4249
Age	0.1470	-0.0238 to 0.3177	0.0916
Year of data collection	0.1450	-0.4190 to 0.7090	0.6143
Regions	0.3198	0.0444 to 0.5951	0.0228
Sample size	-0.2518	-0.4488 to -0.0549	0.0122

Table S4 meta-regression analyses for point prevalence of SPT-positive FA in China

Covariate	Meta-regression estimate	95% CI	P value
Gender	-	-	-
Age	-	-	-
Year of data collection	-0.0769	-0.5605 to 0.4066	0.7552
Regions	0.0737	0.0089 to 0.1385	0.0258
Sample size	-0.3904	-0.5366 to -0.2443	<.0001

Table S5 meta-regression analyses for point prevalence of OFC-positive FA in China

Covariate	Meta-regression estimate	95% CI	P value
Gender	-	-	-
Age	-1.7615	-2.3663 to -1.1567	<.0001
Year of data collection	0.3143	-0.4947 to 1.1232	0.4464
Regions	0.2009	-0.1757 to 0.5774	0.2958
Sample size	-0.4497	-0.9318 to -0.0324	0.0425

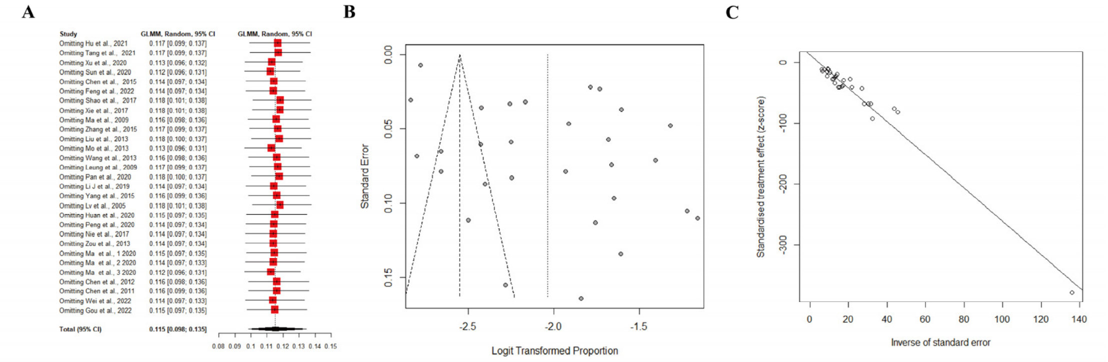

Figure S6 Sensitivity Analysis (A), funnel plot (B) and Egger's tests (C) for the lifetime prevalence of self-reported FA.

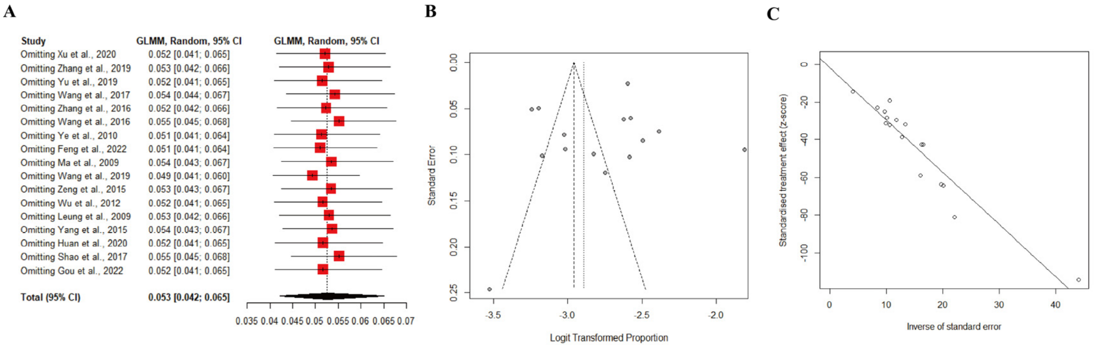

Figure S7 Sensitivity Analysis (A), funnel plot (B) and Egger's tests (C) for the lifetime prevalence of self-reported physician-diagnosed FA.

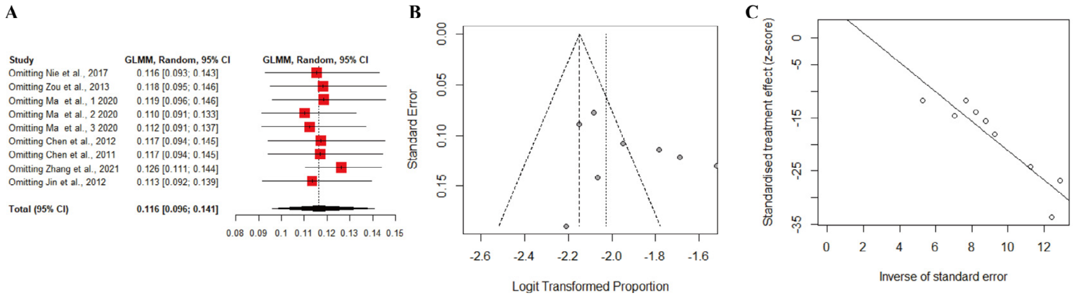

Figure S8 Sensitivity Analysis (A), funnel plot (B) and Egger's tests (C) for the point prevalence of SPT-positive FA.

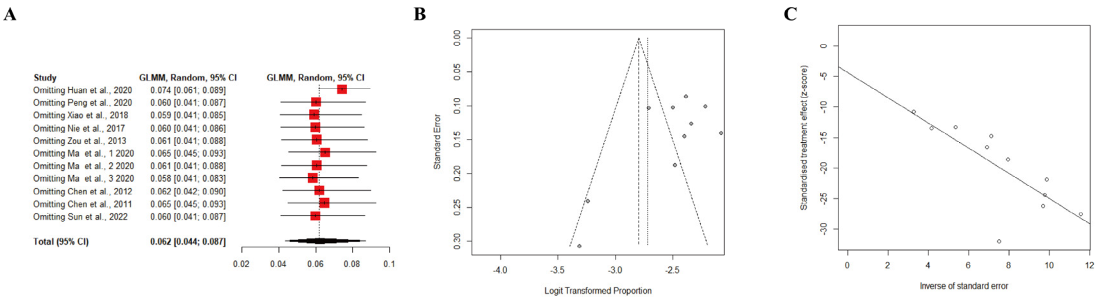

Figure S9 Sensitivity Analysis (A), funnel plot (B) and Egger's tests (C) for the point prevalence of OFC-positive FA.
